# Media Exposure to COVID-19 Predicted Acute Stress: A Moderated Mediation Model of Intolerance of Uncertainty and Perceived Social Support

**DOI:** 10.3389/fpsyt.2020.613368

**Published:** 2021-02-10

**Authors:** Xiangcai He, Yu Zhang, Meng Chen, Jihong Zhang, Weixing Zou, Yu Luo

**Affiliations:** ^1^School of Psychology, Guizhou Normal University, Guiyang, China; ^2^Chengdu Medical College, Chengdu, China; ^3^Xingyi Normal University for Nationalities, Xingyi, China

**Keywords:** COVID-19, media exposure, acute stress, intolerance of uncertainty, perceived social support

## Abstract

**Background:** Previous studies have found that disaster-related media exposure could predict acute stress responses. However, few studies have investigated the relationship between media exposure to COVID-19 and acute stress, and less is known about the mechanisms that translate media exposure to COVID-19 into acute stress. The current study explored the impact of media exposure to COVID-19 on acute stress, and examined the mediating role of intolerance of uncertainty (IU) and the moderating role of perceived social support (PSS).

**Methods:** A total of 1,483 Chinese participants (*M*_*age*_ = 27.93 years, *SD* = 8.45) completed anonymous online questionnaires regarding media exposure to COVID-19, IU, PSS, and acute stress during the COVID-19 outbreak in China.

**Results:** Media exposure to COVID-19 was positively related to acute stress, and IU partially mediated this relationship. The direct effect of media exposure to COVID-19 on acute stress, and the relationship between IU and acute stress, were both moderated by PSS. The impacts of both media exposure to COVID-19 and IU on acute stress were stronger for individuals with low PSS.

**Limitations:** This study collected data in a shorter timeframe, and no assessments occurred during the follow-up, which may prevent us from detecting the changes of the relationships between variables over time. Meanwhile, the self-report method limited the validity of the data due to subjective reporting bias.

**Conclusions:** These findings contribute to a better understanding of how and when pandemic-related media exposure affects acute stress, and provide new perspectives for the prevention to reduce psychological problems following traumatic events.

## Introduction

COVID-19, as a novel Coronavirus was first reported in Wuhan, China, and has rapidly spread into a global pandemic, causing huge numbers of hospitalizations and deaths (1, 2). The Chinese government executed preventative and control measures, including the lockdown of cities, travel bans, and home quarantine, to curb the spread of the virus (3, 4). During the COVID-19 outbreak, the public had a great need for the latest information about COVID-19 from the media to make clear of the situation and protect their health (5, 6). However, the over-reliance on media can cause long term and repeated exposure to the pandemic, which may put the public under psychological distress.

Previous empirical studies have found that media-based indirect exposure to disaster-related events was linked to poor psychological outcomes (7–10). Meanwhile, some studies also indicated that pandemic-related media exposure was positively associated with stress-related symptoms, such as anxiety, depression and worry (5, 6, 11, 12). One study even showed that media exposure was more closely correlated with acute stress than direct exposure (13). Therefore, media exposure to COVID-19 may be an important factor contributing to individuals' acute stress responses. However, less is known about the mechanisms that translate media exposure to COVID-19 into acute stress responses.

Some research suggested that media-related consumption was positively related to intolerance of uncertainty (IU) (14), and IU could lead to poor mental health (15–17). Thus, IU may mediate the relationship between media exposure to COVID-19 and acute stress. According to the stress-buffering model, perceived social support (PSS) may buffer individuals from the adverse effects of stressful events (18). Numerous empirical studies indeed revealed that PSS could moderate the relation between traumatic experiences or stress situations and their influences on people (19–21). Therefore, PSS may affect the relationship between media exposure to COVID-19 and acute stress. To this end, the present study attempted to investigate the relationship between media exposure to COVID-19 and acute stress, and to explore the mechanisms underlying the association by testing the mediating effect of IU and the moderating effect of PSS. The findings would advance our understanding of how and when media exposure to COVID-19 could impact acute stress.

## Theoretical Background and Hypotheses

### Media Exposure to COVID-19 and Acute Stress

According to the risk factor model of the post-traumatic stress response, disaster-related exposure is the primary factor affecting the physical and mental health after traumatic events (22–24). Being one of the disaster-related exposure, disaster-related media exposure can also lead to negative mental health outcomes (9, 25, 26). For instance, Yeung et al. (7) found that frequent exposure to distressing media information could predict PTSD symptoms several months after indirect exposure to the 2008 Wenchuan Earthquake. More importantly, a meta-analysis also demonstrated that media exposure to disasters or large-scale violence had far-reaching effects on poor psychological consequences (27).

Acute stress response refers to a series of physiological and psychological reactions, which is usually triggered by a stressful and life-threatening event (28). Previous empirical research has confirmed the relation between disaster-related media exposure and acute stress responses (10, 29, 30). For example, accumulated evidence indicated that frequently engaging with trauma-related media contents could extend acute stress experiences and increase stress-related symptoms following the Boston Marathon bombings (9, 10, 13). The COVID-19 pandemic, as a public health event, was featured by its rapid transmission, uncertainty about future, considerable mortality rate and serious impacts (31). Facing such an unpredictable and uncontrollable stressful event, the general public are under unprecedented pressure and are experiencing severe psychological distress, including COVID-19-related acute stress responses (32, 33). Correspondingly, some research has also found that the COVID-19 pandemic could induce acute stress responses among the public (33–35). The stressful experiences from either the outbreak itself or the subsequent government responses to the outbreak (e.g., lockdown, travel restrictions) occurred in a very short time period following the COVID-19 outbreak, which may lead to COVID-19-related acute stress responses (28). Besides, the ongoing perceived threats, inconsistent information and uncertainty about the future, accompanied by the pandemic may constitute a risk for mental health (36). When faced with the ambiguous situation and continued threats induced by COVID-19 pandemic, individuals tend to consume information form media to guide them (33). However, media coverage about COVID-19 may amplify the perception of risk, and lead to an exacerbation of stress-related symptoms (5, 6). Therefore, it can be inferred that pandemic-related media exposure could predict COVID-19-related acute stress responses.

Moreover, emotional contagion model indicates that negative emotions can be contagious to each other in crisis events (37, 38). Accordingly, widespread media coverage about disasters may extend the boundary of disaster itself and disseminate passive emotions among the population, thereby increasing psychological distress (39). In fact, the mere exposure of distressing media content is sufficient to provoke negative emotions (5, 6, 40, 41). During the COVID-19 outbreak, media coverage usually contained numerous stress-inducing contents, such as rumors, misrepresentation, and fear messages, especially media-based graphic images (e.g., diagnosed patients with ventilators), all of which would result in huge psychological stress on the public. Thus, it is reasonable that pandemic-related media exposure can promote the formation and development of COVID-19-related acute stress responses. Based on the theoretical and empirical grounds, we hypothesized that media exposure to COVID-19 would be positively correlated with acute stress (Hypothesis 1).

### The Mediating Role of Intolerance of Uncertainty

IU is defined as a relatively broad construct representing cognitive, emotional, and behavioral reactions to uncertainty in everyday life situations, which can be seen as a dispositional tendency (42, 43). According to uncertainty reduction theory, individuals with high IU tend to seek information about the potential threat to reduce anxiety and uncertainty after disasters (44). However, seeking information via the media may backfire when individuals are exposed to disaster-related media content, thereby exacerbating their distress and uncertainty (10, 14). Meanwhile, IU is in general sustained by the associated perception of uncertainty, and the uncertainty comes largely from uncertain situations and life events (43, 45). Given that many aspects of life were full of uncertainty due to the COVID-19 outbreak, pandemic-related media exposure can be seen as an important source of uncertainty. Thus, IU may also emerge in response to “uncertain” media exposure related to COVID-19. Indeed, a few studies have indicated that media-related consumption was positively associated with IU. For example, a meta-analysis showed that increased mobile phone penetration and Internet usage were positively correlated to the rising IU levels (46). Furthermore, broad evidence has showed that IU can be changed by a series of experimental manipulations, in which the uncertainty about the outcome of events was manipulated to induce high or low degrees of IU (47–49). Therefore, we inferred that media exposure to COVID-19 was positively related to IU.

Moreover, IU plays a significant role in the development and maintenance of distress (16, 50). There is increasing evidence to support that IU is closely associated with mental health problems. For instance, ample empirical evidence has shown that IU was a risk factor for affective disorders, such as generalized anxiety disorder (51), obsessive-compulsive disorder (52), major depressive disorder (53). Similarly, some studies have also demonstrated that IU was highly linked with anxiety, depression and worry (17, 54, 55). Furthermore, previous research has also found that IU was related to elevated post-traumatic stress symptoms (PTSS) (56–58). Individuals with high IU are prone to respond negatively to uncertain or ambiguous situations, which may lead to negative psychological responses over time (58, 59). Hence, it is reasonable to infer that IU could affect acute stress. Taken together, we speculated that IU may act as a mediating role between media exposure to COVID-19 and acute stress (Hypothesis 2).

### The Moderating Role of Perceived Social Support

Although disaster-related media exposure may increase the risk of acute stress through IU, it seems impossible that all individuals would experience an equivalent level of acute stress. PSS may moderate the effect of pandemic-related media exposure on acute stress.

PSS refers to an individual's confidence that sufficient support can be available during times of need (60). It can help individuals manage stressful life events by providing a sense of feeling valued and accepted and by prompting appropriate coping responses (18). Several studies suggested that social support was negatively associated with passive emotions, such as anxiety, depression and stress (61–63). According to the stress-buffering model, PSS can buffer individuals from the passive impacts of stressful events (18, 64). As such, individuals with high levels of PSS may present better psychological adjustment (65). Numerous empirical studies have supported this model. For instance, some studies found social support had a potential moderating effect in the relationship between trauma exposure and psychological health outcomes, such as depression and PTSD (66, 67). The risk-buffering hypothesis also holds that one protective factor can mitigate the association between environmental risk factors and negative outcomes (68). Therefore, we inferred that PSS may moderate the relationships between media exposure to COVID-19 and IU, as well as between media exposure to COVID-19 and acute stress.

Moreover, PSS may buffer the negative effects of psychological distress (18, 68). Some research has found that social support could attenuate the relationships between personal risk factors and health outcomes and behaviors (69–71). For example, it was found that PSS moderated the relation between depression and adolescent problematic smartphone use (72), and the relation between psychological insecurity and depression (73). IU is, understandably, a personal risk factor that may cause negative psychological outcomes (e.g., anxiety, depression) (54, 55). Therefore, PSS may act as a moderator in the relationship between IU and acute stress. To some extent, PSS can be seen as a protective factor for stress-related outcomes (74–76), and may contribute to enhancing individuals' internal mental resources (77). As a result, individuals perceiving more social support would be less likely to have psychological problems in response to stressful events or other psychological distress (78, 79). Based on the theoretical views and empirical evidence, we deduced that PSS would moderate the direct and indirect relations between media exposure to COVID-19 and acute stress (Hypothesis 3).

### The Present Study

**Figure 1:**
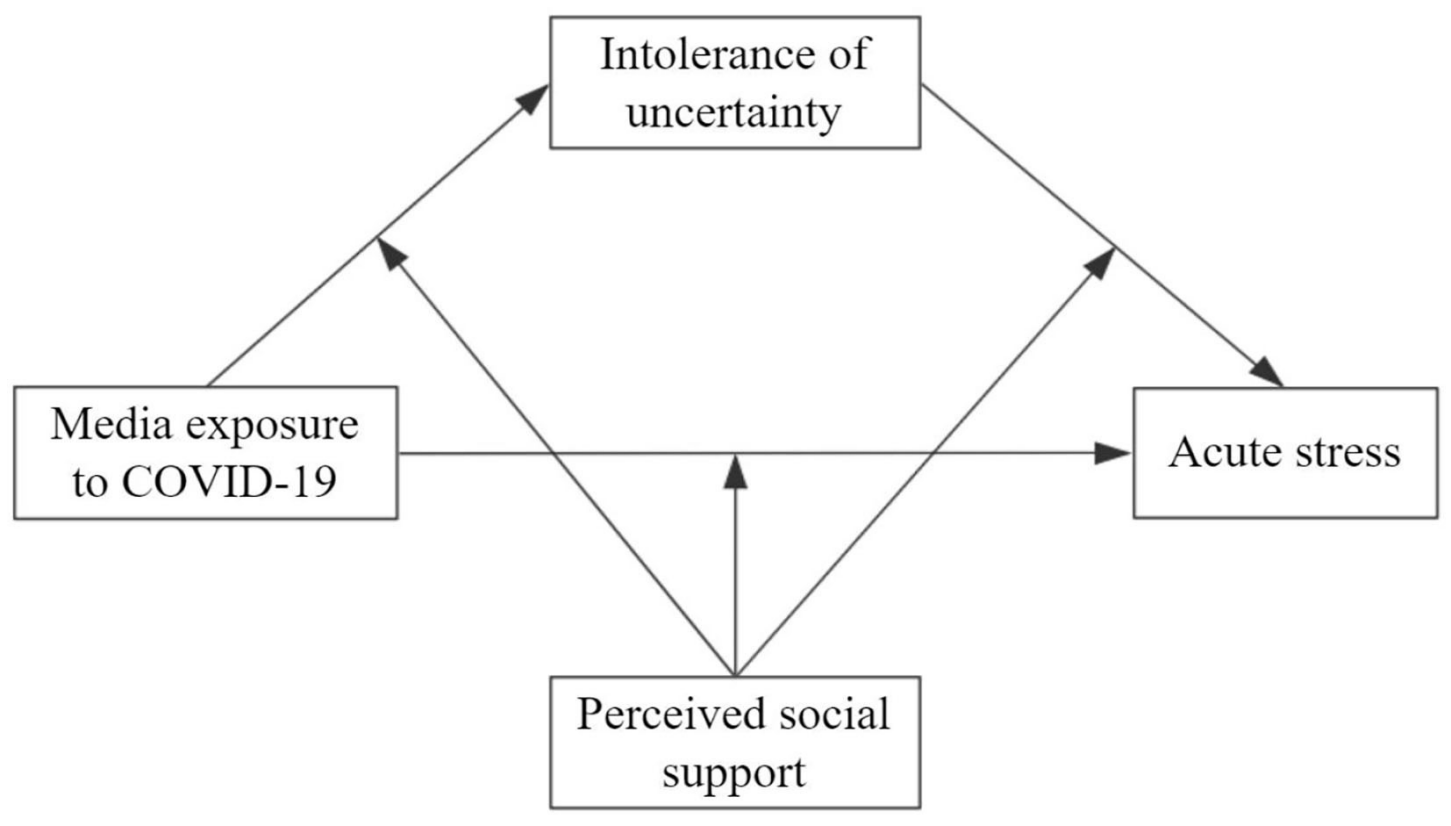
The proposed moderated mediation model.

The present study aimed to examine the impact of media exposure to COVID-19 on acute stress and its underlying mechanisms. First, we examined whether media exposure to COVID-19 would directly affect acute stress. Second, we tested the mediating role of IU in the relation between media exposure to COVID-19 and acute stress. Third, we tested whether the direct and indirect relations between media exposure to COVID-19 and acute stress through IU would be moderated by PSS. Therefore, we proposed a moderated mediation model (see Figure 1).

## Methods

### Participants and Procedure

This survey was conducted from February 7 to February 28, 2020, during the COVID-19 outbreak in China. Participants were required to finish Internet-based questionnaires via social media (WeChat, Tencent). A total of 1,626 participants from 32 provinces or political areas participated in our research. The final sample consisted of 1,483 participants after removing participants who gave uniform answers to all items in the questionnaire and those who were directly exposed to COVID-19 (e.g., close contacts, confirmed cases). Among the participants, 466 (31.42%) were males and 1,017 (68.58%) were females, with a mean age of 27.93 years (*SD* = 8.45; range: 18–87 years), and 932 (62.85%) were single. Nearly half of respondents lived in city (46.66%), and more than half of participants were undergraduate (55.02%). Detailed demographic characteristics are presented in Table 1. All participants signed an electronic informed consent prior to their participation, and they could withdraw at any time if they wished. All procedures performed in this study involving human participants were in accordance with the 1964 Helsinki declaration and its later amendments or comparable ethical standards.

**Table 1:** Demographic characteristics (*n* = 1,483).

**Variables**	**Group**	***N***	**%**
Gender	Male	466	31.42
	Female	1,017	68.58
Age	18–25 years	759	51.18
	26–44 years	651	43.90
	45 years and above	73	4.92
Marital status	Single	932	62.85
	Married	524	35.33
	Divorced or widowed	27	1.82
Place of residence	City	692	46.66
	Town	277	18.68
	Village	514	34.66
Education	High school and below	263	17.73
	Undergraduate	816	55.02
	Graduate and above	404	27.24

### Measures

#### Media Exposure to COVID-19

Media Exposure Questionnaire (MEQ) was developed to test media exposure to COVID-19 following previous research (13, 14). Nine items were used to assess the media exposure to COVID-19 by asking participants how many hours per day (0–24 h) they spent engaged with information about COVID-19 from the nine most common media sources separately (e.g., television, online news, social media). An example item is “How many hours per day did you spend watching TV to know about COVID-19 in the latest week.” Total media exposure scores were calculated based on the accumulated continuous number of hours across types of media, with higher scores indicating higher levels of media exposure to COVID-19. The Cronbach's α in this study was 0.82.

#### Intolerance of Uncertainty

Intolerance of Uncertainty Scale-12 (IUS-12) is a 12-item self-report scale that assesses reactions and desired control over ambiguous or uncertain situations (80). The measure uses a 5-point scale scored from 1 (strongly disagree) to 5 (strongly agree). The total scores can range from 12 to 60, with higher scores indicating more serious IU. The Cronbach's α in current study was 0.88.

#### Perceived Social Support

Perceived social support was tested by Perceived Social Support Scale (PSSS) (81). The PSSS is a 12-item self-report scale, and each item uses a 7-point scale (1 = strongly disagree; 7 = strongly agree). The total scores can range from 12 to 84, with higher scores indicating better social support the participants perceived. In this study, the Cronbach's α was 0.94.

#### Acute Stress

Stanford Acute Stress Reaction Questionnaire (SASRQ) is usually used to measure acute stress and acute stress disorders (ASD) (82). The Chinese version of SASRQ was revised by Jia and Hou (83) through standard translation and back-translation procedure. Many empirical results have showed that the Chinese version of SASRQ has a good reliability and validity (84–86). In present study, some items were modified to ensure that the scale could be suitable to assess COVID-19-related acute stress responses by reference to previous research (9, 10, 13). An example item is “The COVID-19 pandemic made it difficult for me to perform work or other things I needed to do.” The SASRQ is a self-report questionnaire with 30 items including dissociation (10 items), reexperiencing of trauma (six items), avoidance (six items), anxiety and hyperarousal (six items), and impairment in functioning (two items). The measure uses a 6-point scale scored from 0 (not experienced) to 5 (very often experienced). The total scores can range from 0 to 150, with higher scores indicating higher levels of acute stress. The Cronbach's α in current study was 0.95.

### Data Analysis

In this study, all statistical analyses were performed using SPSS 25.0. First, a factor analysis was used to test common method biases. Second, descriptive statistics and Pearson correlations were calculated among the study variables. Third, independent *t*-test and one-way ANOVA were used to compare the differences of study variables in gender, age and marital status. Next, we used Model 4 of the PROCESS macro for SPSS to examine the mediating effect of IU (87). Finally, Model 59 of the PROCESS macro was used to test the moderating effects of PSS in the direct and indirect relationships between media exposure to COVID-19 and acute stress (87). The bootstrapping method (5,000 bootstrapping samples) with 95% confidence intervals (CIs) was conducted to detect the significance of the effects (87). All study variables, except gender and marital status, were standardized in Model 4 and Model 59 before data analyses. Since previous studies reported that gender, age and marital status could influence psychological health following traumatic events (29, 88, 89), we added gender, age and marital status as control variables in the models.

## Results

### Common Method Bias Test

Given that the data were obtained by self-report questionnaires, we conducted a Harman's single factor test to examine the common method biases (90). The results indicated that 10 factors with eigenvalues > 1 were extracted, which explained 62.28% of the total variance. The first principal factor explained 24.75% of the variance. These results showed that no common method bias existed in current study.

### Descriptive Statistics and Correlation Analyses

Means, standard deviations and correlations between main variables are provided in Table 2. Media exposure to COVID-19 was positively correlated with IU (*r* = 0.17, *p* < 0.001) and acute stress (*r* = 0.26, *p* < 0.001), and the Hypothesis 1 was supported. IU was positively correlated with acute stress (*r* = 0.35, *p* < 0.001). However, PSS was negatively correlated with IU (*r* = −0.10, *p* < 0.001) and acute stress (*r* = −0.24, *p* < 0.001).

**Table 2:** Descriptive statistics and intercorrelations between variables (*n* = 1,483).

**Variables**	***M* ±*SD***	**1**	**2**	**3**	**4**
Media exposure to COVID-19	6.98 ± 5.54	1			
Intolerance of uncertainty	32.89 ± 8.41	0.17^***^	1		
Perceived social support	62.35 ± 13.84	−0.02	−0.10^***^	1	
Acute stress	22.37 ± 21.34	0.26^***^	0.35^***^	−0.24^***^	1

*** *p < 0.001*.

### Comparison of Study Variables on Gender, Age and Marital Status

As shown in Table 3, *t*-tests showed that there were significant gender differences in PSS (*t* = −4.30, *p* < 0.001) and acute stress (*t* = −2.02, *p* < 0.05). Females reported higher levels of both PSS and acute stress than males. One-way ANOVAs indicated that age and marital status had significant effects on PSS (both *p* < 0.01). Individuals aged 26–44 and married people had higher levels of PSS.

**Table 3:** Comparison of study variables on gender, age and marital status.

**Variables**	***N***	**MEC**	***t/F***	**IU**	***t/F***	**PSS**	***t/F***	**AS**	***t/F***
		***M±SD***		***M±SD***		***M±SD***		***M±SD***	
**Gender**									
Male	466	6.85 ± 5.59	−0.63	33.46 ± 8.92	1.78	60.01 ± 14.55	−4.30^***^	20.72 ± 21.30	−2.02^*^
Female	1017	7.04 ± 5.52		32.62 ± 8.16		63.43 ± 13.38		23.13 ± 21.33	
**Age**									
18–25 years	759	7.08 ± 5.73	0.32	33.12 ± 8.09	2.24	61.26 ± 13.85	4.99^**^	23.62 ± 21.73	2.95
26–44 years	651	6.92 ± 5.40		32.82 ± 8.64		63.58 ± 13.78		21.27 ± 21.00	
45 years and above	73	6.61 ± 4.80		30.96 ± 9.47		62.74 ± 13.57		19.25 ± 19.70	
**Marital status**									
Single	932	7.08 ± 5.72	0.57	33.00 ± 8.27	0.69	61.46 ± 13.68	7.22^**^	23.09 ± 21.51	2.03
Married	524	6.85 ± 5.29		32.77 ± 8.50		64.13 ± 13.62		20.92 ± 20.85	
Divorced or widowed	27	6.22 ± 4.23		31.19 ± 11.43		58.81 ± 19.60		25.52 ± 24.44	

*MEC, Media exposure to COVID-19; IU, Intolerance of uncertainty; PSS, Perceived social support; AS, Acute stress. t/F, t or F*,

* *p < 0.05*,

** *p < 0.01*,

*** *p < 0.001*.

### Testing for Mediating Effect

In Hypothesis 2, we deduced that IU would mediate the relationship between media exposure to COVID-19 and acute stress. The hypothesis was examined with Model 4 of the PROCESS macro after controlling for gender, age and marital status (87). As Table 4 shows, media exposure to COVID-19 was positively associated with IU [β = 0.17, *t* = 6.60, *p* < 0.001, 95% CI = (0.12, 0.22)], and IU was positively associated with acute stress [β = 0.32, *t* = 13.13, *p* < 0.001, 95% CI = (0.27, 0.36)]. Moreover, when the mediator (IU) was included in the model, media exposure to COVID-19 was also positively associated with acute stress [β = 0.20, *t* = 8.43, *p* < 0.001, 95% CI = (0.16, 0.25)]. This indicated that IU partially mediated the relationship between media exposure to COVID-19 and acute stress. The bootstrapping results also indicated that the conditional indirect effect of media exposure to COVID-19 on acute stress through IU was significant [indirect effect = 0.05, Boot *SE* = 0.009, Boot 95% CI = (0.036, 0.073)]. The mediation effect accounted for 21.38% of the total effect.

**Table 4:** Testing the mediation effect of intolerance of uncertainty on acute stress.

**Predictors (IV)**	**Model 1**			**Model 2**			**Model 3**			**Model 4**		
	**(DV: Acute stress**)			**(DV: Acute stress)**			**(DV: IU)**			**(DV: Acute stress)**		
	**β**	***SE***	***t***	**β**	***SE***	***t***	**β**	***SE***	***t***	**β**	***SE***	***t***
Gender	0.10	0.06	1.83	0.09	0.05	1.74	−0.12	0.06	−2.19^*^	0.13	0.06	2.58^**^
Age	−0.05	0.04	−1.19	−0.04	0.04	−1.17	−0.08	0.04	−2.19^*^	−0.02	0.04	−0.48
Marital status	0.00	0.07	0.01	0.01	0.07	0.12	0.08	0.07	1.08	−0.02	0.07	−0.24
MEC				0.26	0.03	10.23^***^	0.17	0.03	6.60^***^	0.20	0.02	8.43^***^
IU										0.32	0.02	13.13^***^
*R^2^*	0.01			0.07			0.03			0.17		
*F*	2.41			28.10^***^			13.18^***^			59.56^***^		

*IV, Independent variable; DV, Dependent variable; MEC, Media exposure to COVID-19; IU, Intolerance of uncertainty*.

* *p < 0.05*,

** *p < 0.01*,

*** *p < 0.001*.

### Testing for Moderated Mediation

To test moderated mediation (Hypothesis 3), we adopted Model 59 of the PROCESS macro for SPSS after controlling for gender, age and marital status (87). As presented in Table 5, the interaction between media exposure to COVID-19 and PSS had a significant predictive effect on acute stress [β = −0.08, *t* = −3.32, *p* < 0.001, 95% CI = (−0.12, −0.03)], but not on IU [β = −0.02, *t* = −0.83, *p* > 0.05, 95% CI = (−0.07, 0.03)]. The interaction between IU and PSS had a significant predictive effect on acute stress [β = −0.07, *t* = −3.40, *p* < 0.001, 95% CI = (−0.10, −0.03)]. The results suggested that PSS moderated the relationships between media exposure to COVID-19 and acute stress, and between IU and acute stress.

**Table 5:** Testing the moderated mediation effects of media exposure to COVID-19 on acute stress.

**Predictors (IV)**	**Model 1 (DV: IU)**			**Model 2 (DV: Acute stress)**		
	**β**	***SE***	***t***	**β**	***SE***	***t***
Gender	−0.10	0.06	−1.83	0.17	0.05	3.35^***^
Age	−0.08	0.04	−2.04^*^	−0.01	0.03	−0.23
Marital status	0.08	0.07	1.11	−0.01	0.06	−0.08
MEC	0.17	0.03	6.47^***^	0.20	0.02	8.39^***^
PSS	−0.08	0.03	−3.22^**^	−0.21	0.02	−9.25^***^
MEC × PSS	−0.02	0.03	−0.83	−0.08	0.02	−3.32^***^
IU				0.29	0.02	12.34^***^
IU × PSS				−0.07	0.02	−3.40^***^
*R^2^*	0.04			0.23		
*F*	10.74^***^			54.65^***^		

*IV, Independent variable; DV, Dependent variable; MEC, Media exposure to COVID-19; IU, Intolerance of uncertainty; PSS, Perceived social support*.

* *p < 0.05*,

** *p < 0.01*,

*** *p < 0.001*.

**Figure 2:**
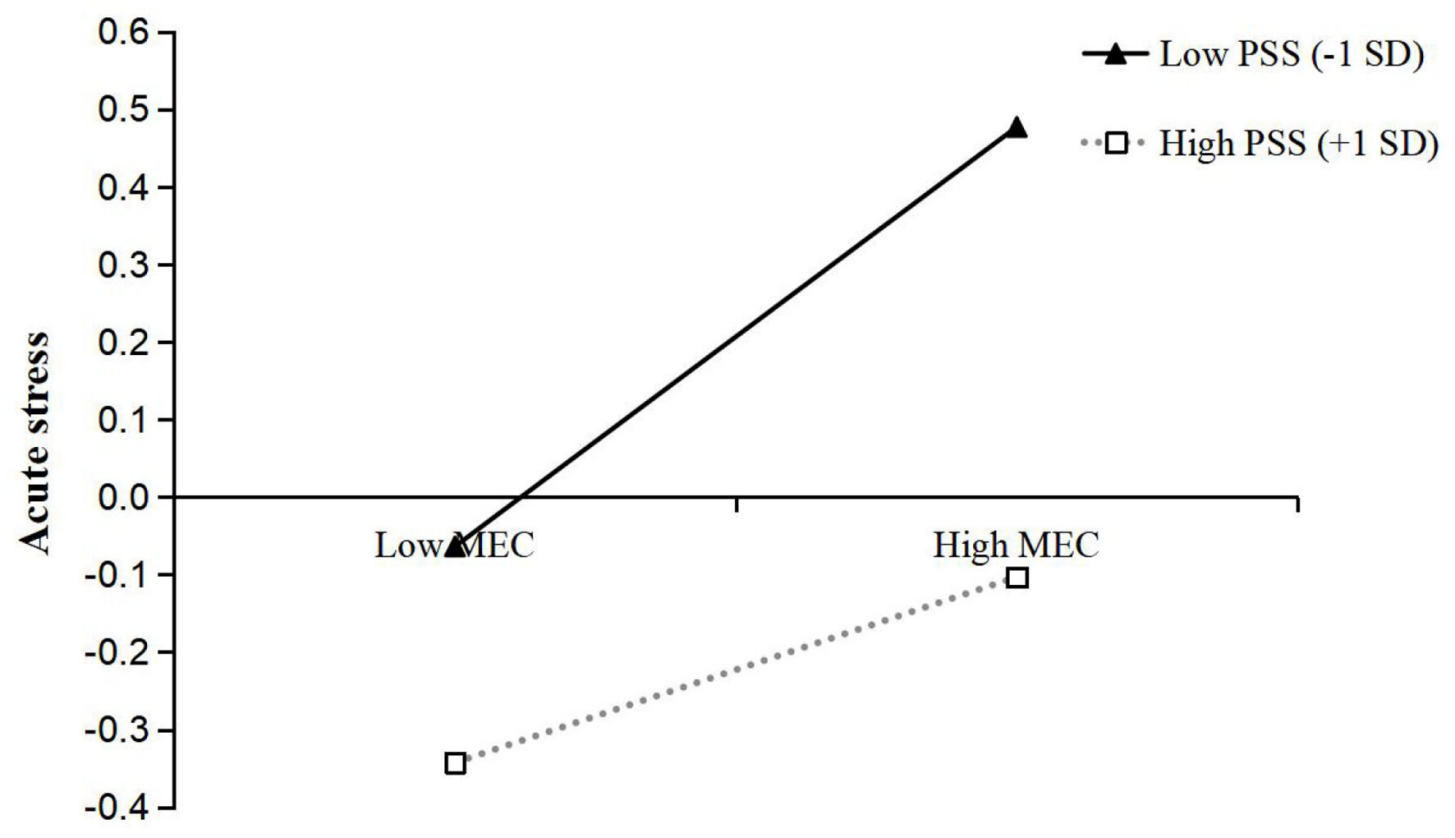
The interaction between media exposure to COVID-19 and perceived social support on acute stress. MEC, Media exposure to COVID-19; PSS, Perceived social support.

**Figure 3:**
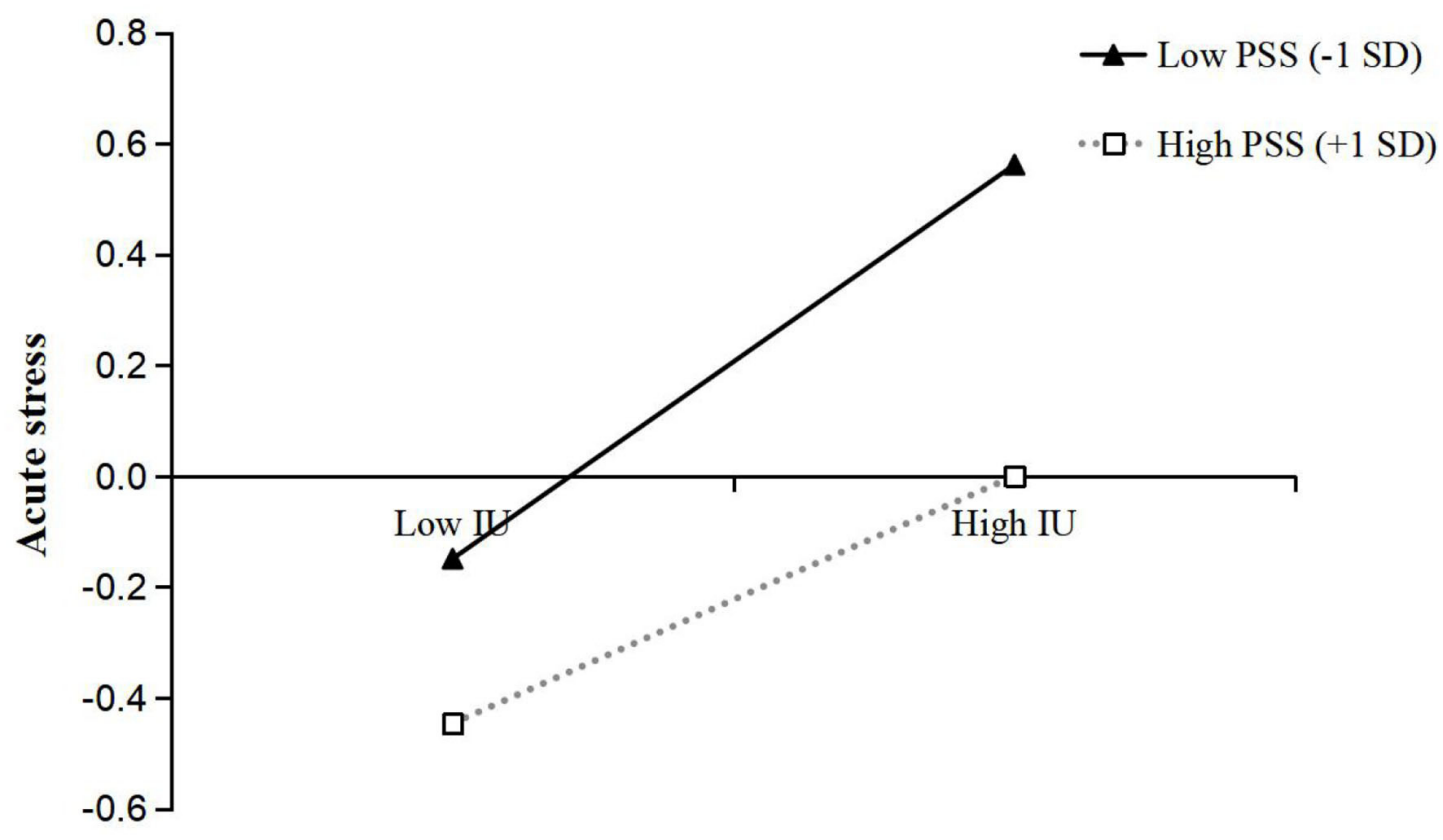
The interaction between intolerance of uncertainty and perceived social support on acute stress. IU, Intolerance of uncertainty; PSS, Perceived social support.

To better interpret the moderating effects of PSS, we examined the simple effects of both media exposure to COVID-19 on acute stress and IU on acute stress, at different levels of PSS (1 *SD* below the mean and 1 *SD* above the mean). Simple slope tests showed that the association between media exposure to COVID-19 and acute stress was stronger for individuals with low PSS (β_*simple*_ = 0.27, *t* = 8.59, *p* < 0.001) than for individuals with high PSS (β_*simple*_ = 0.12, *t* = 3.57, *p* < 0.001) (see Figure 2). Similarly, the association between IU and acute stress was stronger for individuals with low PSS (β_*simple*_ = 0.36, *t* = 12.20, *p* < 0.001) than for individuals with high PSS (β_*simple*_ = 0.22, *t* = 7.04, *p* < 0.001) (see Figure 3).

Moreover, we further examined whether the moderated direct and indirect effects of media exposure to COVID-19 on acute stress were statistically significant. First, the moderated direct effect showed that the association between media exposure to COVID-19 and acute stress was stronger for individuals with low PSS [β = 0.27, *t* = 8.59, *p* < 0.001, 95% CI = (0.21, 0.33)] than for individuals with high PSS [β = 0.12, *t* = 3.57, *p* < 0.001, 95% CI = (0.05, 0.19)]. Second, the bootstrapping results indicated that the indirect effect of media exposure to COVID-19 on acute stress via IU was moderated by PSS [the index of moderated mediation = −0.01, Boot *SE* = 0.004, Boot 95% CI = (−0.020, −0.004)]. The indirect effect of media exposure to COVID-19 on acute stress via IU was stronger for individuals with low PSS [indirect effect = 0.06, Boot *SE* = 0.011, Boot 95% CI = (0.040, 0.084)] than for individuals with high PSS [indirect effect = 0.04, Boot *SE* = 0.008, Boot 95% CI = (0.023, 0.055)]. In addition, the pairwise contrasts between conditional indirect effects (Effect1 minus Effect2) were all significant: Contrasts effect 1 (0.05–0.06) = −0.01, Boot *SE* = 0.004, Boot 95% CI = (−0.020, −0.004); Contrasts effect 2 (0.04–0.06) = −0.02, Boot *SE* = 0.008, Boot 95% CI = (−0.040, −0.008); Contrasts effect 3 (0.04–0.05) = −0.01, Boot *SE* = 0.004, Boot 95% CI = (−0.020, −0.004). In sum, these results indicated that PSS moderated the relationship between media exposure to COVID-19 and acute stress via IU.

## Discussion

In current study, we investigated the influence of media exposure to COVID-19 on acute stress during the COVID-19 outbreak in China, and built a moderated mediation model with IU as a mediating variable and PSS as a moderating variable. Results showed that media exposure to COVID-19 could directly affected acute stress, which supported previous studies that pandemic-related media exposure could lead to stress-related responses (5, 6, 11, 12). Moreover, this study further extended previous research by confirming that media exposure to COVID-19 could affect acute stress indirectly through the mediator of IU, and PSS moderated the relationships between media exposure to COVID-19 and acute stress, as well as between IU and acute stress.

### Comparison of Perceived Social Support and Acute Stress on Demographic Variables

The demographic variable tests on PSS showed that there were significant differences in gender, age and marital status. In particular, the females, the age group of 26–44 years and being married had higher levels of PSS than other groups. Actually, the differences of PSS in the demographic variables of gender, age, marital status are controversial in previous studies against the background of COVID-19 outbreak. For example, Zmete and Pak (91) found the differences of PSS only in marital status but not in gender and age. Contrarily, another study suggested that there were significant differences of PSS in gender and age (36). Therefore, further research is warranted to explore the differences of PSS in the demographic variables. Moreover, we found that females had higher levels of acute stress than males during the COVID-19 outbreak, which supported the most previous studies demonstrating that females generally have more serious psychological symptoms than males following disaster-related events (88, 92). One possible explanation is that as a special group with delicate perception and emotional vulnerability, females are more susceptible to negative outcomes following disasters, thus experiencing higher acute stress. Furthermore, females are vulnerable to multiple stresses in that they are often more sensitive to the guarantee of family stability in China, which may render females more prone to psychological problems during the pandemic.

### Media Exposure to COVID-19 Predicted Acute Stress

The present study discovered that media exposure to COVID-19 was positively correlated with acute stress, even after controlling for demographics. That is, individuals engaging in more pandemic-related information were more likely to show higher acute stress. Our results supported the risk factor model of the post-traumatic stress response (23, 24), suggesting that pandemic-related media exposure was a potential risk factor for mental health. Meanwhile, this further indicated that trauma-related media exposure could predict negative psychological outcomes in different traumatic events (e.g., natural disasters, man-made accidents, public health emergencies). In addition, our results were in line with emotional contagion model (37, 38). This may suggest that emotional contagion is an interactive process between individuals, and the negative emotions induced by COVID-19 pandemic could be contagious to each other. As a result, individuals with more media exposure to COVID-19 were more vulnerable to acute stress.

Furthermore, our findings echoed the previous empirical studies, which stated that disaster-related media exposure was predictably related to acute stress (9, 10, 13). Besides, the present study further supported recent research suggesting that media exposure to COVID-19 could result in stress-related symptoms (5, 6, 11). In the period of COVID-19 outbreak in China, the rapid spread of pandemic caused social isolation of an entire nation, and people also had a great craving for information to figure out the situation and to reduce potential risks and uncertainties. In this situation, media became the main source of pandemic-related information for the majority of people in China. However, prolonged and uncontrolled media exposure could reinforce rumination and intrusive thoughts, activate fear circuitry (13, 93), and enhance autonomic activation and affecting physiologic systems (94–96), thus leading to the increase of acute stress.

### The Mediating Role of Intolerance of Uncertainty

As predicted, IU partially mediated the relationship between media exposure to COVID-19 and acute stress. Therefore, IU may be not only an outcome of media exposure to COVID-19, but also a predictor of acute stress. To our knowledge, this is the first study that tests the mediating effect of IU in the relation between media exposure and acute stress following stressful events.

For the first path of the mediation process, we found that media exposure was positively linked to IU, which coincided with one prior study (14). Media coverage usually contains ambiguous, exaggerated and even dramatic information, which may lead to more information-seeking behaviors aimed at reducing uncertainty and relieving discomfort. However, these information-seeking behaviors could provide new entries to exposure to more pandemic-related information by all kinds of media, in turn causing people to experience more uncertainty. That is, pandemic-related media exposure could provide necessary psychological basis for the generation of IU. Besides, given that COVID-19 is a highly contagious virus without effective treatment and adequate protective materials (2), people with frequent media exposure to COVID-19 are more likely to hold a negative expectation for the future and thus cannot tolerate uncertainty. The findings also supported prior studies revealing that IU could be subject to change in response to uncertainty information or scenes (47–49). Moreover, given that individuals high in IU are more likely to seek information from media to reduce uncertainty, future research is needed to explore the influence of IU on media exposure related to stressful events.

For the second path of the mediation process, this study indicated that IU was positively related to acute stress, which supported the previous research showing that IU could lead to negative psychological outcomes (54, 97, 98). There are two possible explanations for this finding. First, individuals with higher levels of IU may display an exaggerated perception of threat and engage in increased avoidance following a traumatic event due to the uncertainty (57, 80, 99). They usually evidence a greater likelihood to interpret uncertain information as unacceptable and threatening (100, 101). Thus, those high in IU may display increased acute stress. Second, IU, as a tendency to response negatively to uncertain situations and events, essentially reflects the worry about the uncertainty in the future (59). And repeated experiencing such feeling may also contribute to other stress-related psychological symptoms, such as anxiety, depression and PTSD (17, 55, 56). Therefore, it is not difficult to explain that IU can affect acute stress.

### The Moderating Role of Perceived Social Support

Our study further found that PSS weakened the associations between media exposure to COVID-19 and acute stress, as well as between IU and acute stress. This means that the influences of both media exposure to COVID-19 and IU on acute stress got weaker when individuals had higher levels of PSS.

First, we found that PSS could moderate the relation between media exposure to COVID-19 and acute stress. As the stress-buffering model (18) suggests, PSS could buffer individuals from the impact of negative situations. Thus, people with high levels of PSS tend to perceive warmth, and get love and help from their family and friends when they encounter stressful life events (89, 102). These supports can contribute to enhancing positive mental resources and self-efficacy to cope with adversity effectively (77). Accordingly, they are less likely to experience acute stress compared with people with low levels of PSS, when indirectly exposing to stressful events. Consistent with previous studies (74, 76, 77), our findings indicated that PSS could be regarded as a protective factor to promote the positive development of mental health, and to help individuals flexibly adapt to adversity. As the media exposure to COVID-19 prolonged, people could suffer continuously increasing acute stress. In this situation, social support is an important protective resource to produce beneficial psychosocial changes and attenuate the detrimental effects of pandemic-related media exposure on acute stress.

Just as PSS could buffer the negative effects of pandemic-related media exposure on acute stress, PSS also moderated the relation between IU and acute stress. The result supported the stress-buffering model and the risk-buffering hypothesis (18, 68), and further indicated that PSS was a critical protective factor in mitigating the passive effects of personal risk factors on mental health. Similarly, this finding was in line with previous research, suggesting that PSS could buffer the negative effects of personal risk factors (70, 71). Therefore, PSS could to some extent protect the public from a series of adverse impacts caused by IU during the COVID-19 outbreak. This means that although IU could produce negative influences on mental health, the individuals who perceived more social support from their families and friends would be less affected by IU during the COVID-19 pandemic. Additionally, individuals with high levels of social support could take full use of coping strategies to deal with psychological distress (78, 79, 103), thus contributing to reducing their vulnerability to acute stress. Therefore, PSS acted as a stress-buffering factor in the second link of the mediation chain.

Contrary to our hypothesis, PSS did not moderate the link between media exposure to COVID-19 and IU. One possible explanation is that the influence of pandemic-related media exposure on IU is direct, fast and stable, and this process is less susceptible to external factors. Hence, more media exposure to COVID-19 was associated with more serious IU regardless of the level of PSS. Meanwhile, this result also revealed that PSS may not always act as a protective factor to reduce IU in uncertain conditions. Some prior studies supported this view of point as well (104, 105). Therefore, further studies are needed to better clarify the role of PSS in the relation between media exposure and IU following stressful events.

### Limitations and Implications

There are several limitations that should be noted. First, the self-report method limited the validity of the data due to subjective reporting bias. Thus, future research could take various measures to obtain more objective and comprehensive information. Second, we collected data in a shorter timeframe, and no assessments occurred during the follow-up, which may prevent us from detecting the changes of the relationships between variables over time. In future research, we could collect data at different stages of the pandemic to examine the temporal stability of these relationships. Third, we only examined the impacts of overall media exposure to pandemic on acute stress, and did not distinguish different media contents or types. Future studies should further explore the associations between different media contents or types and acute stress responses. Fourth, the present study focused on the passive impacts of pandemic-related media exposure on mental health, but neglected its positive effects. Future research could explore the positive implications of media exposure following public health events. Last, given that the COVID-19 pandemic is not a typical traumatic event, the application of the SASRQ in current study may be limited. Thus, further studies are needed to explore the applicability of the SASRQ in the pandemic-related events.

Despite these limitations, the current study has some theoretical and practical implications. First, this study further extends previous research by confirming the mediating role of IU and the moderating role of PSS. This could contribute to a better understanding of how and when pandemic-related media exposure can influence acute stress. Second, our findings revealed that PSS could help protect individuals from the development of acute stress related to IU. This indicates that it is critical to empower social support networks and minimize uncertain situations for the public, thereby reducing their acute stress responses. Third, our study confirmed the negative impacts of media exposure to pandemic, which could remind the public that appropriate use of media is necessary to maintain psychological health during the pandemic. Similarly, governments and relevant agencies should consider implementing the effective prevention and intervention to reduce negative psychological effects following traumatic events.

## Conclusion

In summary, this study found that increased media exposure to COVID-19 was associated with higher acute stress during the COVID-19 outbreak in China. This association was partially mediated by IU. In particular, increased media exposure to COVID-19 was associated with higher IU, which in turned was associated with higher acute stress. Moreover, PSS can buffer the relationships between media exposure to COVID-19 and acute stress, as well as between IU and acute stress. Specifically, the effect of media exposure to COVID-19 on acute stress was stronger for individuals with low levels of PSS. Similarly, the effect of IU on acute stress was stronger for individuals with low levels of PSS.

## Data Availability Statement

The original contributions presented in the study are included in the article/Supplementary Files, further inquiries can be directed to the corresponding author/s.

## Ethics Statement

The studies involving human participants were reviewed and approved by the Ethical Committee of Guizhou Normal University. All participants provided electronic informed consent prior to their participation.

## Author Contributions

XH designed the research and wrote up the manuscript. YZ analyzed data and wrote up the original draft. MC performed the research. JZ designed the structure and performed the calculations. WZ reviewed literature and revised manuscript. YL reviewed manuscript and supervised the project. All authors contributed to the article and approved the submitted version.

## Conflict of Interest

The authors declare that the research was conducted in the absence of any commercial or financial relationships that could be construed as a potential conflict of interest.
